# Marriage protects against temporomandibular joint disorders: A cross-sectional study

**DOI:** 10.1097/MD.0000000000045246

**Published:** 2025-10-24

**Authors:** Jilun Liu, Shuning Li, Wei Yang

**Affiliations:** a Department of Oral and Maxillofacial Surgery, The Second Hospital of Hebei Medical University, Shijiazhuang, Hebei, China.

**Keywords:** marital status, reproductive status, temporomandibular disorders (TMD), temporomandibular joint (TMJ)

## Abstract

The negative impact of psychological factors on temporomandibular disorders (TMD) has attracted widespread attention. However, the association between marriage as an essential psychological influence and TMD has never been reported. The authors sought to examine the correlation between marriage and TMD. We performed a case-control research with 105 adult patients who met the inclusion criteria during the study period and 114 matched controls who were matched to the patients from our hospital. An investigation was conducted using weighted multivariable and logistic regression analyses to examine the autonomous correlation between marriage history and TMD. Additionally, subgroup analysis and interaction testing were conducted. Conditional logistic regression analysis revealed that, in the fully adjusted model, marriage history was associated with a significantly lower risk of temporomandibular disorders (TMD) (OR = 0.14, 95% CI: 0.03–0.65, *P* = .0126). Gender significantly modified this association (*P* for interaction = .0017), with married men showing an extremely low prevalence of TMD (OR = 0.00, 95% CI: 0.00–0.07, *P* = .0001). After marriage, the risk of TMD decreased, reaching its lowest level among individuals with 1 child. However, as the number of children increased, the incidence of TMD gradually rose and could eventually exceed the premarriage level. Subgroup analyses and curve-fitting methods supported this nonlinear relationship between the number of children and TMD risk. Marriage appears to protect against temporomandibular joint disorders, with the lowest risk observed in individuals with 1 child. However, the incidence of TMD increases as the number of children rises. Further studies are needed to explore the factors contributing to the increased risk of TMD following childbearing.

## 1. Introduction

TMD refer to conditions that cause facial and head discomfort. These illnesses are linked to problems with the jaw, temporomandibular joint, and muscles used for chewing.^[1]^ According to a clinical evaluation conducted in 2015, the most common areas where pain was described were the muscles used for chewing and the temporomandibular joint.^[2]^ In 73% of instances, the pain was a combination of muscle discomfort and joint pain, whereas in 23% of cases, it was only muscle-related pain.^[2]^ Similar to other musculoskeletal problems such as persistent low back pain, fibromyalgia, and headache, chronic painful TMD cannot be attributed to physical factors alone. The etiology of TMD has shifted significantly from a purely mechanistic basis to one that is complex and includes both biological and psychological factors.^[3,4]^

Psychosocial factors are closely associated with TMD. Studies show a bidirectional relationship between TMD and psychological distress, particularly depression and anxiety, within the biopsychosocial framework.^[5,6]^ Stress and parafunctional habits such as bruxism may contribute to pain through neuroimmune mechanisms involving inflammatory mediators.^[7,8]^ Psychological interventions,^[9]^ including internet-based programs,^[10]^ have shown potential in reducing pain and disability. Moreover, chronic TMD-related dysfunction is multidimensional, encompassing pain interference, jaw limitations, negative affectivity, catastrophizing, and heightened pain sensitivity.^[11]^

Several variables impact individuals’ mental well-being, including life stress, work stress, sleep quality, and marital status. Psychological variables have a significant impact on the development of TMD. Most previous studies have focused on gender, age, and psychological stress and have not addressed the impact of marriage history on TMD. For the first time, we focus on the effects of marriage history on TMD.

## 2. Methods

### 2.1. Study design and sample

We studied patients suffering from temporomandibular joint abnormalities treated surgically at our hospital’s Department of Oral and Maxillofacial Surgery from June 1, 2023 to May 31, 2024. All patients received TMJ nuclear magnetic resonance imaging evaluation, and only patients with complete medical record information were included in the study.

The diagnostic criteria of the patients were uniform: All magnetic resonance imaging results were irreversible anterior displacement of the TMJ. Conservative treatment was ineffective. Clinical symptoms were evident, including popping of the TMJ, pain, and limitation of mouth opening. Two physicians diagnosed all patients.

A total of 219 case files were gathered, with 105 exhibiting TMD, while the remaining 114 were considered controls. The control group was selected from patients without TMD who visited our hospital during the same study period. Controls were matched to cases according to age (±2 years) and gender to minimize potential confounding.

### 2.2. Data collection

A retrospective analysis of medical records was conducted on patients diagnosed with irreversible anterior dislocation of the temporomandibular joint who received treatment in the department. The clinical charts and investigative reports were examined.

The following factors were thoroughly recorded: gender, age, oral malocclusion, marital history, and number of children. The clinical registrations were conducted using the methodology developed by Angle categorization, which has been widely employed in several research.

The physicians responsible for diagnosis were blinded to the patients’ marital and reproductive history during the assessment to reduce bias. All patient data were anonymized prior to analysis, and no identifying information was included. The data used in this study are not publicly available due to institutional policies but can be obtained from the corresponding author upon reasonable request.

### 2.3. Data management and analysis

Information was documented using standardized collecting forms. The analyses were conducted using R version 3.4.3 (R Foundation for Statistical Computing; Vienna, Austria)^[12]^ and Empower software (X&Y Solutions, Inc., Boston).^[13]^ The threshold for statistical significance was established at a *P*-value of <.05. Statistical measures were calculated for all variables. The current study considered the marital history as the variable being investigated, whereas the TMD was regarded as the variable being observed.

## 3. Results

### 3.1. General condition of the patient

This study contained a cohort of 232 individuals who had comprehensive medical records. The key features of these patients are presented in Table 1. The desired age for TMD was 26 years, which was lower than the average age of the general population. TMD was more prevalent in females, with a majority of them experiencing substantial oral malocclusion. Among the patients, 73.3% were unmarried, and 76.2% have never given birth. These results exhibited a substantial deviation from the standard population.

**Table 1:** Baseline characteristics of the population with TMD.

TMD	None	Yes	*P*-value
N	114	105	–
Age	40.6 ± 20.8	26.6 ± 14.6	<.001
Gender			
Female	54 (47.4%)	86 (81.9%)	<.001
Male	60 (52.6%)	19 (18.1%)	
Oral malocclusion			
None	82 (71.9%)	35 (33.3%)	<.001
Yes	32 (28.1%)	70 (66.7%)	
Marriage history			
None	36 (31.6%)	77 (73.3%)	<.001
Yes	78 (68.4%)	28 (26.7%)	
Number of children			
0	40 (35.1%)	80 (76.2%)	<.001
1	38 (33.3%)	3 (2.9%)	
2	31 (27.2%)	18 (17.1%)	
3	5 (4.4%)	4 (3.8%)	

Results in table: Mean + SD/ N (%).

*P*-values: Kruskal–Wallis rank sum test for continuous variables, Fisher exact probability test for count variables with theoretical number <10.

### 3.2. Marriage history and TMD

In the fully corrected model, an 86% reduction in the risk of having TMD was observed in those who were married compared to those who were unmarried, OR = 0.14, 95%CI (0.03, 0.65), *P* = .0126 (Table 2).

**Table 2:** The association between marriage history and TMD.

Exposure	Model 1	Model 2	Model 3
Marriage history			
None	1	1	1
Yes	0.17 (0.09, 0.30) < 0.0001	0.27 (0.10, 0.67) 0.0053	0.14 (0.03, 0.65) 0.0126

Data in table: OR (95% CI) *P*-value.

Outcome variable: TMD; exposure variable: marital history.

Model 1 adjusted for: none, Model 2 adjusted for age, gender, Model 3 adjusts for age, sex, oral malocclusion or not, number of children.

TMD = temporomandibular disorders.

The study findings indicate that the association between TMD and marriage history is strongly influenced by gender (*P* for interaction = .0017). Specifically, the data reveals that married males have an extremely low prevalence of TMD, as shown in Table 3.

**Table 3:** Influence of gender in the relationship between TMD and marital history.

Exposure	Gender	Model 3
Marriage history		
None	Female	1
Yes	Female	0.20 (0.04–1.00) 0.0502
None	Male	0.54 (0.21–1.39) 0.2008
Yes	Male	0.00 (0.00–0.07) < 0.0001
*P* for interaction		.0017

TMD = temporomandibular disorders.

**Figure 1.**
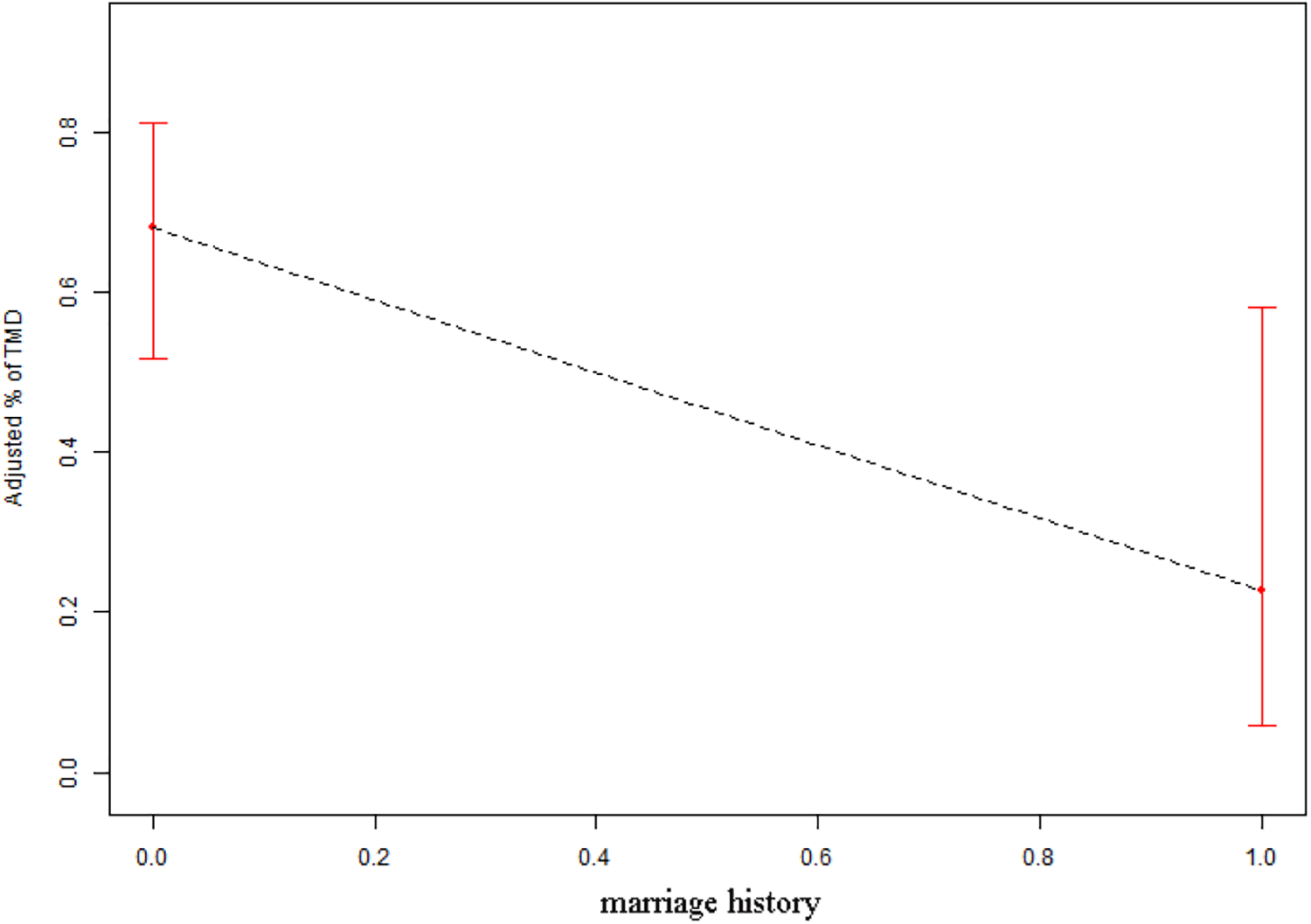
The association between marriage history and TMD. TMD = temporomandibular disorders.

**Figure 2.**
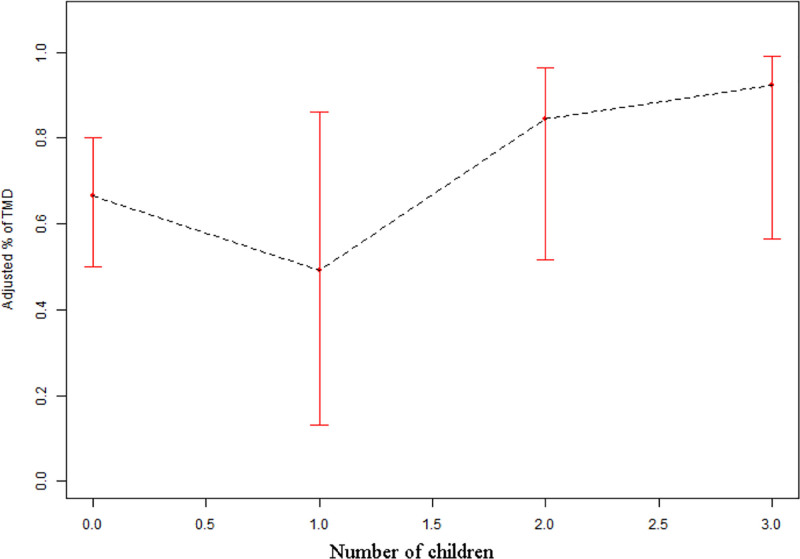
The association between Number of children and TMD. TMD = temporomandibular disorders.

We conducted a curve-fitting analysis to accurately depict the nonlinear correlation between the number of children and TMD. This analysis is illustrated in Figures 1 and 2. The study’s findings indicated a decline in the occurrence of TMD following marriage. Nevertheless, the occurrence of TMD first declined and subsequently rose with the birth of a child, reaching its lowest point when there was just a single child. These results further support our central research question that marital history is protective against TMD, while childbearing introduces a nonlinear effect on TMD risk. Specifically, Figures 1 and 2 demonstrate that marriage reduces TMD risk, but as the number of children increases beyond one, the protective effect diminishes and the incidence gradually rises. This suggests that psychosocial stress associated with raising multiple children may offset the benefits of marriage.

## 4. Discussion

TMD is characterized by discomfort and impaired function in the temporomandibular joint TMJ, jaw, and the associated musculature.^[14]^ TMD refers to the presence of structural abnormalities in the joint. Such disarray can result in synovitis, discomfort over the TMJ, a clicking sound when the disc is realigned, and restricted movement while opening the mouth and locking of the jaw.^[15,16]^

There are many different opinions on the etiology of TMD, and most believe that it is related to psychological factors, occlusal relationships, immune regulation, joint loading, joint anatomy, etc.^[17–22]^ However, there is no standardization of the specific factors that influence it. This study focuses on the social factors of marriage and childbearing. This aspect needs to be more focused on to do a detailed study.

In contemporary culture, there is a growing correlation between mental and physical health, with both exerting a reciprocal impact on one another.^[23]^ Children experiencing TMD discomfort have a greater extent of emotional, somatic, and aggressive behavior compared to healthy control individuals.^[24]^ The pain and discomfort caused by TMJ issues can exacerbate the patient’s psychological distress and impact their sleep quality.^[25]^ Additionally, when the patient experiences high levels of psychological stress, it can further worsen the symptoms of TMD,^[20]^ creating a harmful cycle. TMJ is obviously influenced by individuals’ marital situation, which is a significant factor in mental well-being.

In terms of age, it occurred centrally between 20 and 35 years old. We hypothesize that the possible reasons are:

1. This age group is in puberty, and hormonal regulation often occurs at this stage of life.^[26]^
2. Most patients are students with chronic mental stress, irregular sleep, and long hours of desk work.^[27]^
3. At the peak of jaw development,^[28]^ the patient’s occlusion is rapidly established, and there is a high incidence of malocclusion.^[29,30]^
4. The systemic immune system is gradually improving, and joint contents are gradually becoming more influenced by immune factors.^[31,32]^

The gender prevalence is clearly female, which we believe is due to the fact that adolescent females are highly influenced by hormonal regulation.^[33]^

In our clinical practice, we find that patients often have malocclusion, especially with deep overjet and deep coverage. Due to the underdevelopment of the jaws, the intra-articular pressure is significantly higher in patients with deep overjet than in patients with normal occlusion, which indirectly suggests that increased joint loading is also one of the influencing factors of TMD.^[34]^ It suggests that we should try to break bad habits such as biting hard objects and bruxism at night.^[17]^

In this study, among many influencing factors, we paid special attention to the influence of marital history and the number of children on TMD. Through scientific analysis, our results suggest that marriage at an appropriate age is associated with a lower risk of TMD. This may be due to a combination of factors such as the stabilization of life habits and the reduction of agitation and irritability after marriage; however, we found that the prevalence of TMD increased significantly after childbearing, and most of the children were younger. This may be due to the fact that childbearing itself brings great psychological pressure to the patients, as well as the irregularity of life routine and the steep increase of life pressure due to taking care of the children. This gives us another perspective on recognizing the influencing factors of TMD.

Marital quality has been associated with inflammatory-related conditions, including cardiovascular disease and diabetes.^[35]^ While conflict tends to decrease over the course of marriage, other forms of marital stress, such as emotional disengagement, may still have physiological consequences.^[36]^ Considering the role of inflammation in temporomandibular disorders (TMD), marital stress may represent an important factor influencing TMD risk and severity.

Emerging evidence further suggests that sleep deprivation, parental stress, and related psychosocial factors may play crucial roles in the relationship between TMD and reproductive function. Sleep deprivation can impair male and female fertility by affecting sperm quality, ovarian function, and hormonal balance, while also exacerbating TMD pain through central sensitization, oxidative stress, and pro-inflammatory responses.^[37–39]^ Bruxism may serve as a mediating factor, as fragmented sleep increases systemic stress, potentially disrupting the hypothalamic–pituitary–gonadal axis.^[40,41]^ Additionally, sleep loss may aggravate fertility impairment and TMD-related inflammation through metabolic dysregulation and immune activation.^[42]^ Collectively, these findings indicate that TMD and reproductive function may be interconnected through a complex network involving sleep, psychological stress, bruxism, and oxidative/inflammatory pathways, providing a mechanistic context for the observed post-childbearing increase in TMD prevalence.

This study explains for the first time the influence of marital and childbearing status on TMD, reveals the influencing factors of TMD from another perspective, and fills in the lack of basic information about TMD patients in the northern part of China. Nevertheless, it is necessary to acknowledge the constraints of this study. This study has several limitations. First, the cross-sectional design prevents the establishment of causality between marital and reproductive status and TMD. Second, although we adjusted for some relevant variables, other potential confounding factors, including socioeconomic status, education, lifestyle behaviors, and mental health, were not fully accounted for. Third, the sample size was relatively small and drawn from a single hospital, which may limit generalizability. Finally, retrospective data collection may be subject to information bias. Future studies with larger, multicenter cohorts and prospective designs are needed to validate these findings and further explore the mechanisms underlying these associations.

## 5. Conclusion

Marriage is associated with a lower likelihood of temporomandibular joint disorders (TMD), and the incidence of TMD is lowest among individuals with only 1 child. However, the incidence of TMD appears to increase as the number of children rises. Future longitudinal and multicenter studies are warranted to confirm these associations, explore the underlying mechanisms, and evaluate the impact of marital quality, parental stress, and other psychosocial factors on TMD risk.

## Acknowledgments

The authors thank all the oral and maxillofacial surgeons for their contribution to making the information easily accessible.

## Author contributions

**Conceptualization:** Shuning Li.

**Data curation:** Shuning Li.

**Formal analysis:** Shuning Li.

**Funding acquisition:** Shuning Li.

**Investigation:** Shuning Li.

**Methodology:** Shuning Li.

**Project administration:** Shuning Li.

**Resources:** Shuning Li.

**Software:** Shuning Li.

**Supervision:** Shuning Li.

**Validation:** Wei Yang.

**Visualization:** Wei Yang.

**Writing – original draft:** Jilun Liu.

**Writing – review & editing:** Jilun Liu.

## References

[1] Singh BP Singh N Jayaraman S. Occlusal interventions for managing temporomandibular disorders. Cochrane Database Syst Rev. 2024;9:CD012850. 39282765 10.1002/14651858.CD012850.pub2 PMC11403706

[2] Durham J Newton-John TRO Zakrzewska JM. Temporomandibular disorders. BMJ. 2015;350:h1154. 25767130 10.1136/bmj.h1154

[3] Slade GD Ohrbach R Greenspan JD. Painful temporomandibular disorder: decade of discovery from OPPERA studies. J Dent Res. 2016;95:1084–92. 27339423 10.1177/0022034516653743 PMC5004239

[4] Cayrol T van den Broeke EN Gerard E. Chronic temporomandibular disorders are associated with higher propensity to develop central sensitization: a case-control study. Pain. 2023;164:e251–8. 36251966 10.1097/j.pain.0000000000002803

[5] Wan J Lin J Zha T. Temporomandibular disorders and mental health: shared etiologies and treatment approaches. J Headache Pain. 2025;26:52. 40075300 10.1186/s10194-025-01985-6 PMC11899861

[6] Zhao R Ye Z Lv X Li Z Xiong X. Imaging brain networks: insights into mechanisms of temporomandibular disorders. J Dent Res. 2025;104:380–8. 39876597 10.1177/00220345241302046

[7] Yi Y Zhou X Xiong X Wang J. Neuroimmune interactions in painful TMD: mechanisms and treatment implications. J Leukoc Biol. 2021;110:553–63. 34322892 10.1002/JLB.3MR0621-731RR

[8] Ettlin DA Napimoga MH Meira E Cruz M Clemente-Napimoga JT. Orofacial musculoskeletal pain: an evidence-based bio-psycho-social matrix model. Neurosci Biobehav Rev. 2021;128:12–20. 34118294 10.1016/j.neubiorev.2021.06.008

[9] Penlington C Bowes C Taylor G. Psychological therapies for temporomandibular disorders (TMDs). Cochrane Database Syst Rev. 2022;8:CD013515. 35951347 10.1002/14651858.CD013515.pub2 PMC9370076

[10] Lam J Svensson P Alstergren P. Internet-based multimodal pain program with telephone support for adults with chronic temporomandibular disorder pain: randomized controlled pilot trial. J Med Internet Res. 2020;22:e22326. 33048053 10.2196/22326 PMC7592067

[11] Miller VE Chen DG Barrett D. Understanding the relationship between features associated with pain-related disability in people with painful temporomandibular disorder: an exploratory structural equation modeling approach. Pain. 2020;161:2710–9. 32639367 10.1097/j.pain.0000000000001976 PMC7669591

[12] Bentzley BS Han SS Neuner S Humphreys K Kampman KM Halpern CH. Comparison of treatments for cocaine use disorder among adults: a systematic review and meta-analysis. JAMA Netw Open. 2021;4:e218049. 33961037 10.1001/jamanetworkopen.2021.8049 PMC8105751

[13] Shuning L Wei Y Xuhui F Jianfeng D Jilun L. Association between lipid accumulation product (LAP) index and self-reported oral health outcomes: a cross-sectional study. Lipids Health Dis. 2025;24:131. 40186201 10.1186/s12944-025-02543-4 PMC11969856

[14] Alshanqiti I Son H Shannonhouse J. Posttraumatic hyperalgesia and associated peripheral sensitization after temporomandibular joint injury in mice. Pain. 2024;166:1597–609. 39715145 10.1097/j.pain.0000000000003498 PMC12168817

[15] Gan Z Zhao Y Wu Y Yang W Zhao Z Zhao L. Three-dimensional, biomimetic electrospun scaffolds reinforced with carbon nanotubes for temporomandibular joint disc regeneration. Acta Biomater. 2022;147:221–34. 35562008 10.1016/j.actbio.2022.05.008

[16] Yan Y Yu L Zhang X. Instantaneous self-recovery and ultra-low detection limit hydrogel electronic sensor for temporomandibular disorders intelligent diagnosis. Nat Commun. 2025;16:839. 39833158 10.1038/s41467-025-55996-7 PMC11747250

[17] Silva TB Ortiz FR Maracci LM. Association among headache, temporomandibular disorder, and awake bruxism: a cross-sectional study. Headache. 2022;62:748–54. 35674092 10.1111/head.14322

[18] Li Y Fang M Niu L. Associations among gastroesophageal reflux disease, mental disorders, sleep and chronic temporomandibular disorder: a case-control study. CMAJ. 2019;191:E909–15. 31427355 10.1503/cmaj.181535 PMC6699946

[19] Kristensen KD Stoustrup P Küseler A Pedersen TK Twilt M Herlin T. Clinical predictors of temporomandibular joint arthritis in juvenile idiopathic arthritis: a systematic literature review. Semin Arthritis Rheum. 2016;45:717–32. 26708936 10.1016/j.semarthrit.2015.11.006

[20] Herpel C Drusko A Schwindling FS Rammelsberg P Tesarz J. Head and neck pain drawing area correlates with higher psychosocial burden but not with joint dysfunction in temporomandibular disorders: a cross-sectional study. J Pain. 2023;24:970–9. 36682594 10.1016/j.jpain.2023.01.010

[21] Olliver SJ Broadbent JM Thomson WM Farella M. Occlusal features and TMJ clicking: a 30-year evaluation from a cohort study. J Dent Res. 2020;99:1245–51. 32660369 10.1177/0022034520936235

[22] Stoustrup P Lerman MA Twilt M. The temporomandibular joint in juvenile idiopathic arthritis. Rheum Dis Clin North Am. 2021;47:607–17. 34635294 10.1016/j.rdc.2021.06.004

[23] Hui TT Garvey L Olasoji M. Perspectives of mental health clinicians on physical health of young people with early psychosis. Int J Ment Health Nurs. 2024;33:649–59. 38012093 10.1111/inm.13268

[24] Al-Khotani A Gjelset M Naimi-Akbar A Hedenberg-Magnusson B Ernberg M Christidis N. Using the child behavior checklist to determine associations between psychosocial aspects and TMD-related pain in children and adolescents. J Headache Pain. 2018;19:88. 30242517 10.1186/s10194-018-0915-6 PMC6755608

[25] Błaszczyk B Waliszewska-Prosół M Smardz J Więckiewicz M Wojakowska A Martynowicz H. Exploring the associations of sleep bruxism and obstructive sleep apnea with migraine among patients with temporomandibular disorder: a polysomnographic study. Headache. 2025;65:242–57. 39740030 10.1111/head.14892 PMC11794979

[26] Pries LK Prachason T Arias-Magnasco A Lin BD Rutten BPF Guloksuz S. Impact of puberty timing, status and oestradiol on psychotic experiences in the context of exposomic and genomic vulnerability to schizophrenia in female adolescents: longitudinal ABCD study. Br J Psychiatry. 2025;1:1–8. 10.1192/bjp.2025.36 40899184

[27] Abe S Kawano F Matsuka Y Masuda T Okawa T Tanaka E. Relationship between oral parafunctional and postural habits and the symptoms of temporomandibular disorders: a survey-based cross-sectional cohort study using propensity score matching analysis. J Clin Med. 2022;11:6396. 36362625 10.3390/jcm11216396 PMC9654264

[28] Oxilia G Menghi Sartorio JC Bortolini E. Exploring directional and fluctuating asymmetry in the human palate during growth. Am J Phys Anthropol. 2021;175:847–64. 33973654 10.1002/ajpa.24293 PMC8360102

[29] Mylonopoulou IM Sifakakis I Berdouses E Kavvadia K Arapostathis K Oulis CJ. Orthodontic status and orthodontic treatment need of 12- and 15-year-old Greek adolescents: a national pathfinder survey. Int J Environ Res Public Health. 2021;18:11790. 34831543 10.3390/ijerph182211790 PMC8620264

[30] Kavaliauskienė A Šidlauskas A Zaborskis A. Relationship between orthodontic treatment need and oral health-related quality of life among 11–18-year-old adolescents in Lithuania. Int J Environ Res Public Health. 2018;15:1012. 29772849 10.3390/ijerph15051012 PMC5982051

[31] Crincoli V Anelli MG Quercia E Piancino MG Di Comite M. Temporomandibular disorders and oral features in early rheumatoid arthritis patients: an observational study. Int J Med Sci. 2019;16:253–63. 30745806 10.7150/ijms.28361 PMC6367523

[32] Kroese JM Volgenant CMC Crielaard W. Temporomandibular disorders in patients with early rheumatoid arthritis and at-risk individuals in the Dutch population: a cross-sectional study. RMD Open. 2021;7:e001485. 33397683 10.1136/rmdopen-2020-001485 PMC7783521

[33] Mauras N Ross J Mericq V. Management of growth disorders in puberty: GH, GnRHa, and aromatase inhibitors: a clinical review. Endocr Rev. 2023;44:1–13. 35639981 10.1210/endrev/bnac014

[34] Tanaka E Koolstra JH. Biomechanics of the temporomandibular joint. J Dent Res. 2008;87:989–91. 18946004 10.1177/154405910808701101

[35] Wilson SJ Syed SU Yang IS Cole SW. A tale of two marital stressors: comparing proinflammatory responses to partner distress and marital conflict. Brain Behav Immun. 2024;119:898–907. 38718908 10.1016/j.bbi.2024.05.003

[36] Schiltz HK Van Hecke AV. Applying the vulnerability stress adaptation model of marriage to couples raising an autistic child: a call for research on adaptive processes. Clin Child Fam Psychol Rev. 2021;24:120–40. 33118107 10.1007/s10567-020-00332-2

[37] Cavalhas-Almeida C Cristo MI Cavadas C Ramalho-Santos J Álvaro AR Amaral S. Sleep and male (in)fertility: a comprehensive overview. Sleep Med Rev. 2025;81:102080. 40153995 10.1016/j.smrv.2025.102080

[38] Li Z Gong S Yu Z. Sleep deprivation impacts the immunological milieu of epididymis leading to low sperm quality in rats. Commun Biol. 2025;8:644. 40263515 10.1038/s42003-025-08091-y PMC12015424

[39] Caetano G Bozinovic I Dupont C Léger D Lévy R Sermondade N. Impact of sleep on female and male reproductive functions: a systematic review. Fertil Steril. 2021;115:715–31. 33054981 10.1016/j.fertnstert.2020.08.1429

[40] Li Y Zhang W Liu M. Imbalance of autophagy and apoptosis induced by oxidative stress may be involved in thyroid damage caused by sleep deprivation in rats. Oxid Med Cell Longev. 2021;2021:5645090. 34545297 10.1155/2021/5645090 PMC8449739

[41] De la Torre Canales G Poluha RL Soares FFC. Who is the patient with resistant myofascial temporomandibular disorders pain? A somatosensory, psychosocial, and genetic characterization. J Headache Pain. 2025;26:98. 40329183 10.1186/s10194-025-02055-7 PMC12054165

[42] Akhlaghi M Kohanmoo A. Sleep deprivation in development of obesity, effects on appetite regulation, energy metabolism, and dietary choices. Nutr Res Rev. 2025;38:4–24. 37905402 10.1017/S0954422423000264

